# Differential Risk of Obstructive Sleep Apnea in Alcoholic Versus Non-Alcoholic Fatty Liver Disease: A Propensity-Matched Cohort Study

**DOI:** 10.3390/medicina61122146

**Published:** 2025-12-01

**Authors:** Ling-Hui Chang, Hui-Cheng Lin, Wen-Che Hsieh, Chao-Yu Hsu

**Affiliations:** 1 Division of Respiratory Therapy, Ditmanson Medical Foundation, Chia-Yi Christian Hospital, Chia-Yi 600, Taiwan; 2 Department of Physical Therapy, Lin Shin Medical Corporation, Lin Shin Hospital, Taichung 408, Taiwan; 3 Department of Chinese Medicine, Ditmanson Medical Foundation, Chia-Yi Christian Hospital, Chia-Yi 600, Taiwan; 4 Department of Nursing, Min-Hwei Junior College of Health Care Management, Tainan 736, Taiwan; 5 Department of Medical Education, Ditmanson Medical Foundation, Chia-Yi Christian Hospital, Chia-Yi 600, Taiwan; 6 Department of Artificial Intelligence and Healthcare Management, Central Taiwan University of Science and Technology, Taichung 406, Taiwan; 7 Department of General Education, National Chin-Yi University of Technology, Taichung 411, Taiwan

**Keywords:** sleep apnea, alcoholic fatty liver disease, non-alcoholic fatty liver disease, TriNetX

## Abstract

*Background and Objectives*: Obstructive sleep apnea (OSA) is a clinically relevant comorbidity in both alcoholic fatty liver disease (AFLD) and non-alcoholic fatty liver disease (NAFLD). However, whether its impact differs between these etiologies remains unclear. This study directly compared OSA risk in patients with AFLD and NAFLD to elucidate its role in disease progression. *Materials and Methods*: We conducted a retrospective cohort study using the TriNetX research network. Adults aged ≥ 20 years with newly diagnosed AFLD or NAFLD between 2006 and 2020 were included. Propensity score matching was applied to balance demographic and clinical covariates. The primary endpoint was incident OSA, assessed at 1-, 2-, 3-, and 5-year intervals, and cumulatively through 28 September 2025. Effect estimates were expressed as relative risk, odds ratio and hazard ratio (HR). *Results*: Before matching, 896,302 NAFLD and 12,694 AFLD patients were identified; after 1:1 PSM, 11,583 patients remained in each group with balanced baseline characteristics. NAFLD patients consistently demonstrated higher OSA risk. Post-matching, OSA incidence became significantly elevated from year 2 onward (HR at 2 years = 1.764) and persisted at 3 years (HR = 2.078), 5 years (HR = 1.950), and cumulative follow-up (HR = 1.940). *Conclusions*: NAFLD confers nearly double the long-term OSA risk compared with AFLD. These findings support longitudinal OSA screening and targeted risk reduction strategies in NAFLD populations.

## 1. Introduction

Alcohol-associated fatty liver disease (AFLD) represents a significant and growing public health challenge worldwide, with evidence highlighting both high prevalence and increasing severity. In U.S. adolescents and young adults, more than half of individuals with excessive alcohol use demonstrated suspected AFLD, and over 6% showed advanced fibrosis, underscoring the early onset of clinically relevant disease [1]. Large-scale epidemiologic data further indicate worsening trends in adults: analysis from three national U.S. databases revealed that while overall alcoholic liver disease prevalence remained stable, advanced fibrosis and cirrhosis-related complications rose sharply, with hospitalizations for alcoholic cirrhosis increasing by 32.8% between 2007 and 2014 and transplant listings for alcoholic liver disease rising by 63.4% [2]. Longitudinal cohort studies also confirm the prognostic severity of AFLD. Compared with individuals without fatty liver disease, patients with AFLD and intermediate-to-high fibrosis scores exhibited markedly elevated risks of liver-related mortality, with hazard ratio (HR) exceeding 59 in advanced stages [3]. Collectively, these findings emphasize that AFLD is not only prevalent across age groups but also strongly associated with progressive liver damage and death, highlighting the urgent need for targeted prevention, early detection, and alcohol use reduction strategies.

Nonalcoholic fatty liver disease (NAFLD) has emerged as the most common chronic liver disease worldwide, closely linked to obesity, diabetes mellitus (DM), and metabolic syndrome. Meta-analyses estimate the global prevalence at approximately 25–32%, with regional variation ranging from over 40% in South-East Asia and the Americas to lower rates in Africa [4,5]. Recent systematic reviews confirm an upward trend, with prevalence rising from 27.9% in 2000–2010 to over 31% in 2011–2021 [6]. The incidence of NAFLD is also substantial. A large meta-analysis including over 1.2 million individuals reported an incidence of 46.1 per 1000 person-years, with higher risk observed in males, smokers, and overweight or obese individuals [7]. Similarly, global estimates suggest an incidence of 46.2 per 1000 person-years, with projections indicating further increases if current trends continue [6]. In addition, NAFLD-related mortality is estimated at nearly 24 per 1000 person-years, underscoring its severity [6]. NAFLD is a growing global epidemic with rising prevalence and significant progression to cirrhosis, cancer, and death. Public health strategies should prioritize early detection, obesity reduction, and metabolic risk management to curb its impact [6,7].

Obstructive sleep apnea (OSA) is a prevalent disorder characterized by recurrent upper airway collapse and intermittent hypoxia during sleep. Alcohol consumption and NAFLD are both important contributors to chronic liver disease, and recent evidence suggests their relationship with OSA. A meta-analysis demonstrated that alcohol use is positively associated with OSA, with alcohol consumers having a 1.33-fold higher risk of developing OSA compared to non-drinkers [8]. Similarly, growing evidence links OSA with NAFLD. A meta-analysis including over 2200 participants showed that OSA was independently associated with elevated liver enzymes, particularly alanine transaminase, and with histological changes such as steatosis, inflammation and fibrosis [9]. Taken together, these findings highlight OSA as a shared and clinically relevant risk factor in both alcohol-associated and metabolic-related liver diseases. However, the differential impact of OSA between AFLD and NAFLD has not been systematically investigated. Our study aims to address this gap by directly comparing the influence of OSA on patients with AFLD and NAFLD, thereby clarifying its contribution to disease progression across distinct etiologies.

## 2. Materials and Methods

### 2.1. Data Source

This retrospective cohort study utilized data obtained from the TriNetX research network, an extensive international health analytics system integrating electronic health records (EHR) from roughly 179 million individuals across 158 healthcare organizations worldwide. The platform compiles a wide range of clinical information, including patient demographics, diagnostic codes based on the International Classification of Diseases, Tenth Revision, Clinical Modification (ICD-10-CM), therapeutic procedures, and medication prescriptions. To ensure compliance with legal and ethical standards, all datasets are systematically de-identified and anonymized, eliminating any potential for re-identification. Consequently, the use of this database is exempt from institutional review board approval. The federated design of the TriNetX system safeguards strict data security and privacy requirements, while simultaneously supporting large-scale, real-world, population-based observational investigations.

### 2.2. Study Population

The analytical cohort included adults aged 20 years and older who received a new diagnosis of fatty liver disease between 1 January 2006, and 31 December 2020. Patients were classified into two groups according to diagnostic coding: NAFLD (ICD-10-CM: K76.0) and AFLD (ICD-10-CM: K70.0). The index date corresponded to the first recorded diagnosis of fatty liver disease. Individuals with indeterminate sex, pre-existing liver cancer (ICD-10-CM: C22), or a prior history of OSA (ICD-10-CM: G47.33) were excluded. Extracted baseline variables comprised demographic characteristics (age and sex) and clinically pertinent comorbidities, including overweight/obesity (ICD-10-CM: E66), DM (ICD-10-CM: E08–E13), chronic kidney disease (CKD; ICD-10-CM: N18), and depressive episodes (ICD-10-CM: F32). To address confounding and minimize selection bias, propensity score matching was performed using the TriNetX platform’s validated matching algorithm. Age, sex, and the specified comorbidities were incorporated as covariates, yielding a 1:1 matched sample with well-balanced baseline distributions across NAFLD and AFLD cohorts.

### 2.3. Statistical Analysis

The principal study endpoint was the incidence of OSA. Risk assessments were conducted at 1-, 2-, 3-, and 5-year intervals from the index date, as well as across the cumulative observation period ending 28 September 2025. Comparative analyses were undertaken to estimate differential OSA risk between the NAFLD and AFLD cohorts. The effectiveness of propensity score matching was evaluated by calculating standardized differences (Std diff), with values below 0.1 indicating satisfactory covariate balance. Effect estimates were quantified using relative risk (RR), odds ratios (OR), and HR, each reported with corresponding 95% confidence intervals (CI). The Cox proportional hazards model assumes that the HR between comparison groups remains constant throughout the follow-up period. Violation of this assumption may lead to biased or unstable HR estimates. To verify this, we assessed the proportionality and recorded the corresponding *p*-values. This analytic framework ensured robust longitudinal comparisons and minimized potential confounding in assessing OSA risk across disease categories.

## 3. Results

**Figure 1:**
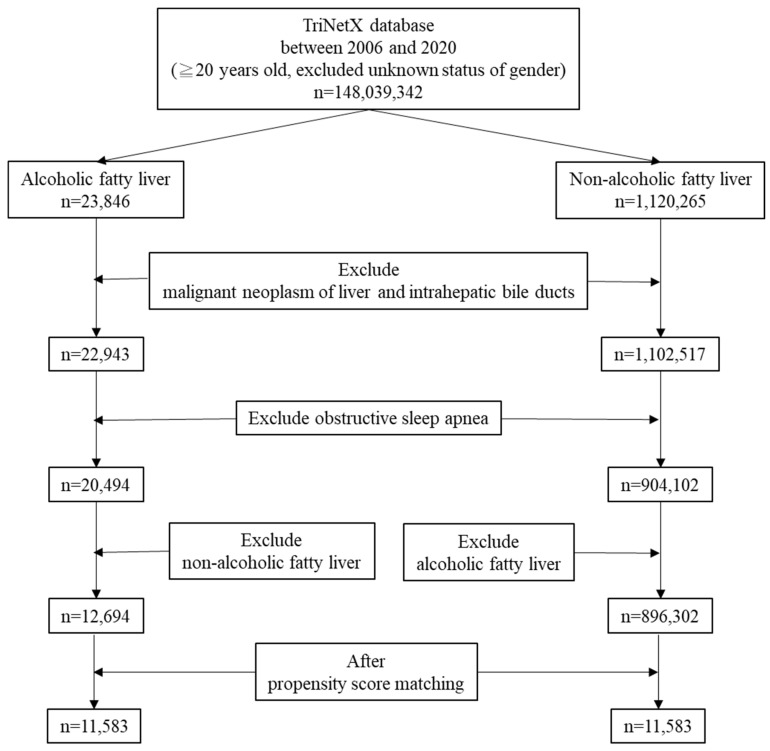
The flowchart of patients’ selection.

The flowchart illustrating patient selection is shown in Figure 1. Table 1 presents the baseline characteristics of patients diagnosed with NAFLD (n = 896,302) and AFLD (n = 12,694) prior to propensity score matching. The mean age was slightly higher in the AFLD cohort compared with the NAFLD group (52.0 ± 13.0 vs. 51.3 ± 16.0 years; *p* < 0.0001). A marked sex imbalance was observed, with women comprising 53.7% of the NAFLD population but only 29.5% of AFLD patients (Std diff = 0.5077). Conversely, men represented 70.5% of the AFLD group compared with 46.3% of NAFLD. Std diff were also noted in comorbidity distribution. DM (19.5% vs. 10.6%; Std diff = 0.2506) and overweight/obesity (18.5% vs. 5.9%; Std diff = 0.3918) were more prevalent in NAFLD. CKD was slightly more frequent among NAFLD (4.4% vs. 3.6%), whereas depressive episodes were comparable between groups.

Table 2 summarizes the baseline characteristics of patients with NAFLD (n = 11,583) and AFLD (n = 11,583) after propensity score matching. Following matching, demographic and clinical characteristics were well balanced between groups. The mean age at index date was identical (51.9 ± 13.0 years in both groups; *p* = 0.9972). Sex distribution was fully matched, with 30.2% female and 69.8% male in each cohort (Std diff < 0.0001). Comorbidities were also comparable: depressive episodes occurred in 13.2% of patients in both groups, DM in 10.3%, overweight/obesity in approximately 6.3%, and CKD in 3.7%. All Std diffs were negligible (<0.01), and all *p*-values were nonsignificant, indicating successful covariate balance. These results confirm that the propensity score matching procedure effectively eliminated baseline disparities, ensuring that subsequent outcome comparisons between NAFLD and AFLD cohorts were not confounded by demographic or comorbidity differences.

Table 3 presents the risk analysis of OSA in patients with NAFLD (n = 896,302) and AFLD (n = 12,694) before propensity score matching. Across all follow-up intervals, NAFLD patients exhibited consistently higher OSA risk compared with AFLD patients. Within 1 year, the risk was 0.244% in NAFLD versus 0.134% in AFLD, with a HR of 1.630 (95% CI: 1.011–2.627). The disparity widened at 2 years (0.729% vs. 0.315%; HR = 2.022, 95% CI: 1.482–2.759) and 3 years (1.411% vs. 0.520%; HR = 2.318, 95% CI: 1.820–2.953). At 5 years, NAFLD patients remained at significantly elevated risk (3.040% vs. 1.197%; HR = 2.086, 95% CI: 1.778–2.446). Cumulative analysis through 28 September 2025 demonstrated an OSA risk of 5.767% in NAFLD compared with 2.300% in AFLD (HR = 2.062, 95% CI: 1.838–2.313). The results showed no significant violations of the proportional hazards assumption across all follow-up periods. The *p*-values for proportionality at 1-, 2-, 3-, and 5-year follow-up, as well as through 28 September 2025, were 0.2165, 0.8089, 0.9652, 0.3062, and 0.2409, respectively.

Table 4 illustrates the risk of OSA among patients with NAFLD (n = 11,583) and AFLD (n = 11,583) after propensity score matching. Following adjustment, NAFLD patients consistently demonstrated higher OSA risk across all follow-up intervals. Within 1 year, the risk was 0.207% for NAFLD versus 0.147% for AFLD, though the HR of 1.289 (95% CI: 0.693–2.400) did not reach significance. At 2 years, the disparity became significant (0.682% vs. 0.345%; HR = 1.764, 95% CI: 1.206–2.580). This trend strengthened at 3 years (1.355% vs. 0.570%; HR = 2.078, 95% CI: 1.559–2.770) and 5 years (3.013% vs. 1.295%; HR = 1.950, 95% CI: 1.610–2.361). Cumulative analysis through 28 September 2025 confirmed sustained excess OSA risk in NAFLD (5.767%) compared with AFLD (2.504%), with HR = 1.940 (95% CI: 1.690–2.227). After propensity score matching, no significant violations of the proportional hazards assumption were detected across any follow-up period. The *p*-values assessing proportionality at 1-, 2-, 3-, and 5-year follow-up, as well as over the full observation period through 28 September 2025, were 0.7202, 0.5512, 0.5847, 0.6414, and 0.7488, respectively.

## 4. Discussion

This represents the first comparative analysis of OSA in NAFLD and AFLD populations. We found that patients with NAFLD have a significantly higher risk of OSA than those with AFLD, both before and after propensity score matching. While the 1-year difference was nonsignificant post-matching, significant disparities emerged from the 2-year follow-up and persisted through 5 years and cumulative analysis. HR consistently approximated 2.0, confirming that NAFLD is associated with a sustained excess OSA risk independent of baseline characteristics.

Evidence from case-control studies demonstrates alcohol’s independent effect on OSA risk. Yang et al. [10] analyzed 793 patients using polysomnography and found that alcohol consumers exhibited over a twofold higher risk of OSA compared with abstainers. The effect was evident in both former and current drinkers, with stronger associations in women, where alcohol intake significantly correlated with elevated apnea–hypopnea index (AHI). These findings suggest both cumulative and sex-specific vulnerabilities. Large-scale population studies further substantiate these results. Ko et al. [11], using data from 11,859 participants in the Korean National Health and Nutrition Examination Survey, reported that alcohol use disorder significantly increased OSA risk (adjusted OR = 2.14). Risk escalation was dose-dependent, correlating with frequency of drinking, binge episodes, and snoring severity. Notably, the unemployed subgroup demonstrated the highest vulnerability (adjusted OR = 2.45), highlighting possible socioeconomic modifiers of alcohol-related OSA burden. Meta-analytic evidence consolidates these associations. Kolla et al. [12], pooling randomized controlled trials, demonstrated that alcohol significantly increased AHI and reduced mean SpO_2_. Effects were more pronounced in individuals with preexisting OSA, where AHI worsened by more than seven events per hour, and in habitual snorers. Similarly, Simou et al. [13] analyzed 21 epidemiological studies and reported a 25% increased RR of OSA among higher alcohol consumers, with stronger effects observed in low- and middle-income countries. Cohort evidence also suggests differential risk by sex and habitual consumption. In the Wisconsin Sleep Cohort, Peppard et al. [14] found that among men, each additional daily drink increased the odds of mild or worse sleep-disordered breathing by 25%. In contrast, minimal to moderate alcohol intake in women was not significantly associated with OSA risk. These results align with Yang et al.’s [10] findings that alcohol-related vulnerability may differ by sex.

Alcohol consumption exacerbates OSA through multiple physiological pathways. As a central nervous system depressant and muscle relaxant, alcohol reduces genioglossal muscle tone, thereby increasing upper airway collapsibility and resistance during sleep [12,13]. Moreover, chronic alcohol use may further damage alveolar type II cells and deplete antioxidant defenses, thereby aggravating hypoxic injury [10]. These mechanisms highlight alcohol as a modifiable determinant that intensifies OSA incidence and severity. These studies provide evidence that alcohol consumption is consistently associated with increased OSA risk across diverse populations, study designs, and analytic methods. The relationship is dose-responsive, particularly pronounced in individuals with alcohol use disorder or preexisting OSA, and may be influenced by socioeconomic context and sex differences. From a public health perspective, alcohol abstinence or reduction emerges as a key preventive and therapeutic measure to mitigate OSA burden.

Across population-based and clinic cohorts, NAFLD is consistently linked with elevated risk of OSA, beyond shared adiposity. The strongest directional evidence comes from a nationwide Korean cohort of 8.1 million adults, in which NAFLD—operationalized by fatty liver index (FLI)—predicted incident, claims-defined OSA over a median 6.3 years. Compared with FLI < 30, participants with FLI 30–60 and ≥60 had higher hazards of developing OSA (adjusted HR 1.15 and 1.21, respectively), and these gradients persisted after adjustment for body mass index (BMI) and abdominal obesity, indicating that hepatic steatosis confers additional OSA risk independent of adiposity [15]. Complementing these data, a nationally representative cross-sectional analysis showed that NAFLD prevalence rose stepwise across STOP-Bang risk strata in both men and women; associations were stronger among individuals with obesity, underscoring effect modification by adiposity while supporting a population-level NAFLD–OSA linkage [16]. Clinic-based studies with objective sleep phenotyping provide pathophysiologic context relevant to NAFLD populations. In a single-center cohort (n = 1065) with polysomnography and abdominal ultrasonography, metabolic dysfunction-associated fatty liver disease (MAFLD) prevalence increased from 58% (no OSA) to 78% (severe OSA); in multivariable models, oxygen desaturation index independently predicted MAFLD, highlighting nocturnal hypoxemia as a salient correlate of liver fat in real-world patients [17]. Similarly, nocturnal hypoxemia metrics (AHI, desaturation burden, time with SpO_2_ < 90%) independently tracked with NAFLD severity after adjustment for BMI and insulin resistance, with hypoxia duration showing the strongest correlation—findings that, from an epidemiologic standpoint, identify OSA-related hypoxemia as a key exposure dimension relevant to NAFLD patients [18]. Among biopsy-characterized individuals enriched for metabolic syndrome, moderate–severe OSA associated with higher odds of hepatic fibrosis independent of obesity, implying that NAFLD patients who harbor OSA are at greater risk of fibrotic progression [19]. A systematic review further concludes that OSA co-occurs with NAFLD more than expected by chance and that severity–severity relationships are common; while continuous positive airway pressure may stabilize hepatic disease in some reports, definitive causal reversal remains uncertain [20].

Clinical data indicates that the principal biological conduit linking OSA with NAFLD is chronic intermittent hypoxia (CIH), coupling cyclical desaturation–reoxygenation to hepatic metabolic injury. CIH generates reactive oxygen species and mitochondrial dysfunction, activates hypoxia-inducible factors and reprograms hepatocellular metabolism toward lipogenesis, thereby promoting steatosis [21,22,23]. Moreover, OSA perturbs the gut–liver axis—by increasing intestinal permeability and altering the gut microbiota—thereby establishing an additional loop that accelerates NAFLD progression [23].

Previous studies have demonstrated associations between obesity [24], DM [25], CKD [26], and depression [27] with the development of OSA. However, in the present study, following propensity score matching to control for obesity, DM, CKD, and depression, patients with NAFLD exhibited a significantly elevated risk of developing OSA compared to those with AFLD, with HR consistently approximating 2.0 beyond 2 years of follow-up. These findings suggest that NAFLD is associated with a sustained excess risk of OSA compared to AFLD, independent of baseline comorbidities and measured confounders.

This retrospective, EHR-based study has several limitations. First, diagnoses were ascertained using ICD-10-CM codes, which are vulnerable to misclassification (over-, or undercoding). Distinguishing NAFLD from AFLD is particularly challenging and any differential error could bias effect estimates in unpredictable directions. However, the codes used to identify NAFLD and AFLD are widely employed in population-based studies and have been shown in prior research to possess reasonable validity for epidemiologic purposes. Although misclassification cannot be fully excluded, these codes represent the standard approach for large-scale observational studies. In addition, large sample size across multiple health systems, which reduces the influence of idiosyncratic coding practices from any single institution. Consistent direction of effect across multiple time points, suggesting that the observed association is robust rather than driven by random coding variability. And, propensity score matching on demographic and metabolic factors, which minimizes differential misclassification associated with comorbidity burden. Second, although TriNetX offers large, structured datasets, it lacks clinical detail such as OSA severity, liver histology or fibrosis stage, and symptom duration, which may affect interpretation. Third, key lifestyle factors and social determinants of health—smoking intensity, diet, physical activity, and socioeconomic context—are incompletely captured; given the established association between smoking and OSA [28], residual confounding is likely. Moreover, detailed information on alcohol consumption (e.g., quantity, frequency, duration, drinking patterns) is not available in the TriNetX database. Although TriNetX aggregates EHR data from participating healthcare organizations, lifestyle-level behavioral data—such as actual alcohol intake, patient-reported drinking history, or validated alcohol-use questionnaires—are not captured within this platform. Therefore, the diagnostic misclassification remains a potential limitation. Because alcohol use is often underreported or incompletely documented in EHR, we acknowledge that the absence of alcohol consumption data may contribute to residual misclassification. Fourth, as an observational analysis, even with 1:1 propensity score matching, unmeasured or imperfectly measured confounders cannot be fully eliminated, and causal inference is limited. Recent advancements in artificial intelligence (AI) have increasingly shaped modern healthcare research. AI-enabled platforms support large-scale data integration, pattern recognition, and real-time analytics, thereby enhancing the generation of real-world evidence [29]. Such systems facilitate efficient cohort identification, risk stratification, and clinical decision support across diverse healthcare settings. If AI were further integrated into platforms like TriNetX—using machine-learning–assisted tools to analyze multi-institutional EHR—it could further strengthen data harmonization and analytical precision. These capabilities would enhance robust and timely investigation of disease associations, including the comparative risks examined in the present study.

Despite these constraints, the breadth of the TriNetX network confers substantial statistical power and enhances generalizability. Within this context, our matched analyses consistently indicate that patients with NAFLD have a significantly higher risk of OSA than those with AFLD, supporting a robust association that appears independent of measured baseline comorbidities. Recent nationwide cohort studies—such as investigations examining the association between statin use and the prognosis of hepatocellular carcinoma after resection [30]—which exemplify how large-scale clinical databases can be leveraged to evaluate risk factors, treatment outcomes, and disease trajectories in hepatology. Highlighting such work underscores the methodological parallels between these studies and our own, and it illustrates the growing potential of real-world evidence platforms to advance liver disease research. Future research may leverage large-scale big-data platforms to incorporate detailed alcohol-consumption metrics, multimodal clinical information, and machine-learning analytics to refine fatty liver phenotyping and elucidate its relationship with incident OSA. Cross-population analyses and interventional studies may further clarify underlying mechanisms and inform strategies to reduce OSA risk in AFLD and NAFLD patients.

## 5. Conclusions

Patients with NAFLD have approximately twice the risk of OSA compared with those with AFLD, with the difference becoming statistically significant from year 2 onward and persisting through 5-year and cumulative follow-up. Clinicians should implement longitudinal OSA screening and risk stratification in NAFLD (e.g., STOP-Bang questionnaire with confirmatory polysomnography when indicated) and concurrently intensify management of modifiable risk factors—weight reduction, smoking cessation, and alcohol moderation—with early referral to sleep-medicine services for high-risk individuals.

## Figures and Tables

**Table 1:** Characteristics of patients with alcoholic and non-alcoholic fatty liver diseases before propensity score matching.

	NAFLD (n, %)	AFLD (n, %)	*p*-Value	Std Diff
	(n = 896,302)	(n = 12,694)		
Age at index (mean ± SD)	51.3 ± 16.0	52.0 ± 13.0	<0.0001	0.0457
Female	481,679 (53.7%)	3743 (29.5%)	<0.0001	0.5077
Male	414,623 (46.3%)	8951 (70.5%)	<0.0001	0.5077
Comorbidities				
Depressive episode	113,141 (12.6%)	1620 (12.8%)	0.6400	0.0042
Diabetes mellitus	175,000 (19.5%)	1349 (10.6%)	<0.0001	0.2506
Overweight and obesity	165,746 (18.5%)	750 (5.9%)	<0.0001	0.3918
Chronic kidney disease	39,062 (4.4%)	456 (3.6%)	<0.0001	0.0392

NAFLD: non-alcoholic fatty liver disease; AFLD: alcoholic fatty liver disease alcoholic; Std diff: standardized difference; SD: standard deviation.

**Table 2:** Characteristics of patients with alcoholic and non-alcoholic fatty liver diseases after propensity score matching.

	NAFLD (n, %)	AFLD (n, %)	*p*-Value	Std Diff
	(n = 11,583)	(n = 11,583)		
Age at index (mean ± SD)	51.9 ± 13.0	51.9 ± 13.0	0.9972	<0.0001
Female	3499 (30.2%)	3499 (30.2%)	1.0000	<0.0001
Male	8084 (69.8%)	8084 (69.8%)	1.0000	<0.0001
Comorbidities				
Depressive episode	1530 (13.2%)	1531 (13.2%)	0.9845	0.0003
Diabetes mellitus	1189 (10.3%)	1188 (10.3%)	0.9827	0.0003
Overweight and obesity	732 (6.3%)	733 (6.3%)	0.9785	0.0004
Chronic kidney disease	434 (3.7%)	434 (3.7%)	1.0000	<0.0001

NAFLD: non-alcoholic fatty liver disease; AFLD: alcoholic fatty liver disease alcoholic; Std diff: standardized difference; SD: standard deviation.

**Table 3:** Risk Analysis of patients with obstructive sleep apnea at 1-, 2-, 3-, and 5-year intervals and cumulative follow-up to 28 September 2025 before propensity score matching.

	Patients with OSA					
	NAFLD (Risk) (n = 896,302)	AFLD (Risk) (n = 12,694)	Risk Ratio (95% CI)	Odds Ratio (95% CI)	Hazard Ratio (95% CI)	*p*-Value for Proportionality
1-year	2183 (0.244%)	17 (0.134%)	1.819 (1.129, 2.930)	1.821 (1.129, 2.935)	1.630 (1.011, 2.627)	0.2165
2-year	6531 (0.729%)	40 (0.315%)	2.312 (1.695, 3.154)	2.322 (1.701, 3.170)	2.022 (1.482, 2.759)	0.8089
3-year	12,643 (1.411%)	66 (0.520%)	2.713 (2.131, 3.453)	2.738 (2.148, 3.489)	2.318 (1.820, 2.953)	0.9652
5-year	27,244 (3.040%)	152 (1.197%)	2.538 (2.166, 2.974)	2.587 (2.203, 3.037)	2.086 (1.778, 2.446)	0.3062
Till 28 September 2025	51,673 (5.767%)	292 (2.300%)	2.506 (2.237, 2.808)	2.598 (2.313, 2.919)	2.062 (1.838, 2.313)	0.2409

OSA: obstructive sleep apnea; NAFLD: non-alcoholic fatty liver disease; AFLD: alcoholic fatty liver disease alcoholic; CI: confidence intervals.

**Table 4:** Risk Analysis of patients with obstructive sleep apnea at 1-, 2-, 3-, and 5-year intervals and cumulative follow-up to 28 September 2025 after propensity score matching.

	Patients with OSA					
	NAFLD (Risk) (n = 11,583)	AFLD (Risk) (n = 11,583)	Risk Ratio (95% CI)	Odds Ratio (95% CI)	Hazard Ratio (95% CI)	*p*-Value for Proportionality
1-year	24 (0.207%)	17 (0.147%)	1.412 (0.759, 2.626)	1.413 (0.759, 2.631)	1.289 (0.693, 2.400)	0.7202
2-year	79 (0.682%)	40 (0.345%)	1.975 (1.351, 2.887)	1.982 (1.354, 2.901)	1.764 (1.206, 2.580)	0.5512
3-year	157 (1.355%)	66 (0.570%)	2.369 (1.786, 3.168)	2.398 (1.796, 3.200)	2.078 (1.559, 2.770)	0.5847
5-year	349 (3.013%)	150 (1.295%)	2.327 (1.925, 2.812)	2.368 (1.952, 2.872)	1.950 (1.610, 2.361)	0.6414
Till 28 September 2025	668 (5.767%)	290 (2.504%)	2.303 (2.012, 2.637)	2.383 (2.071, 2.742)	1.940 (1.690, 2.227)	0.7488

OSA: obstructive sleep apnea; NAFLD: non-alcoholic fatty liver disease; AFLD: alcoholic fatty liver disease alcoholic; CI: confidence intervals.

## Data Availability

This study utilized the TriNetX platform (https://trinetx.com) (accessed on 28 September 2025), a global collaborative network that provides access to de-identified electronic health records for real-world evidence research.
